# Supplementary material for: The interactome of intact mitochondria by cross-linking mass spectrometry provides evidence for coexisting respiratory supercomplexes
Source: Mol Cell Proteomics. 2017 Dec 8;17(2):216–32. doi: 10.1074/mcp.RA117.000470 (PMC5795388; doi:10.1074/mcp.RA117.000470)
Supplement: Supplemental Data [file supp_RA117.000470_133922_0_supp_23978_fzffwf.zip › spectra_annotation/mito_DR_spectra_annotation/365-1-4-1-6-1.pdf]

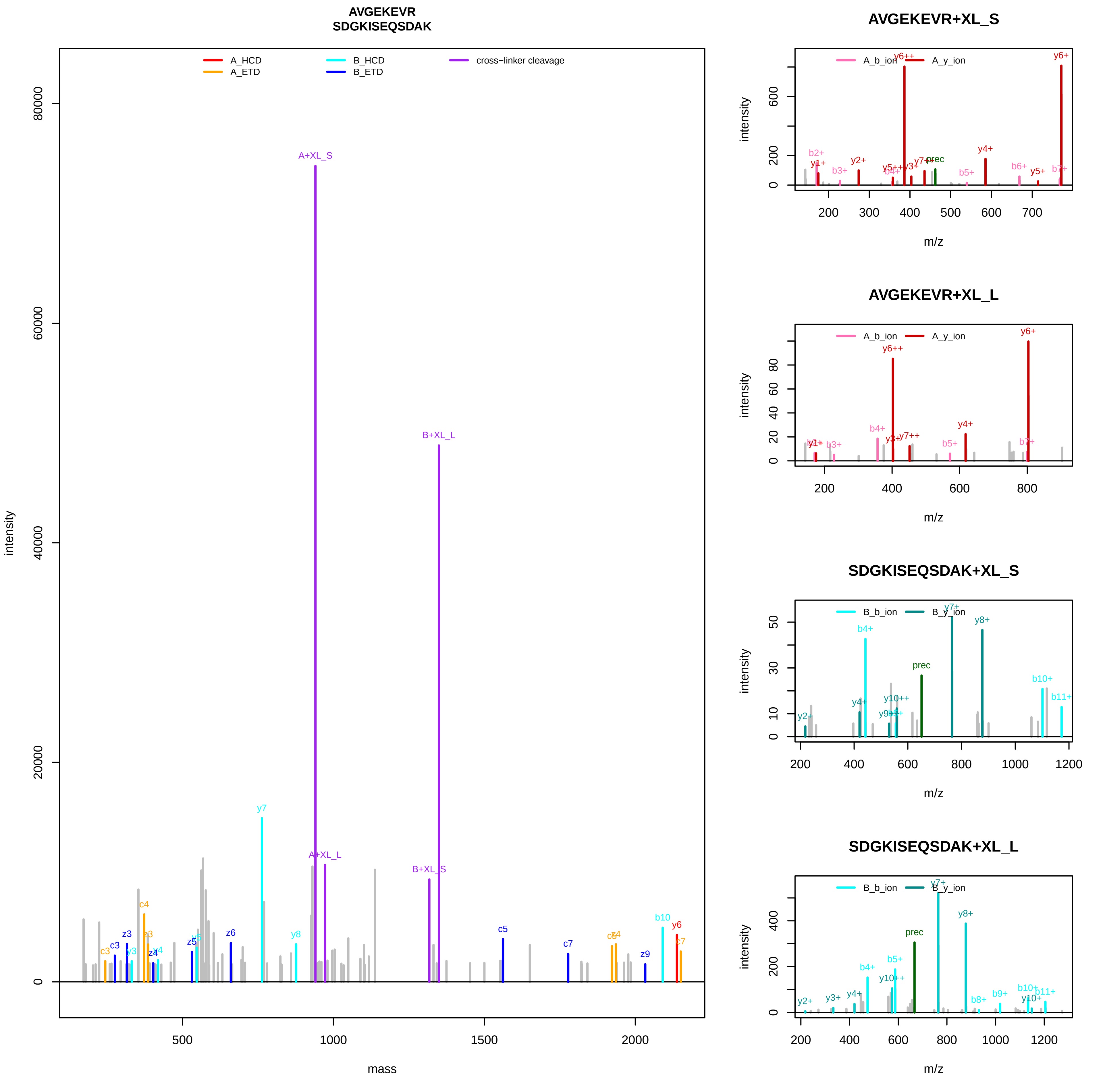
AVGEKEVR
SDGKISEQSDAK
A_HCD
A_ETD
B_HCD
B_ETD
cross−linker cleavage
A+XL_S
B+XL_L
A+XL_L
B+XL_S
intensity
mass
0
20000
40000
60000
80000
500
1000
1500
2000
c3
z3
y3
c4
z4
y4
z5
z6
y7
y8
c5
c7
z9
b10
y6
c7
AVGEKEVR+XL_S
A_b_ion
A_y_ion
b2+
y1+
b3+
y2+
y6++
b4+
y5++
y3+
y7++
prec
b5+
y4+
b6+
y5+
b7+
y6+
m/z
AVGEKEVR+XL_L
SDGKISEQSDAK+XL_S
B_b_ion
B_y_ion
y2+
y4+
b4+
y10++
y9++
prec
y7+
y8+
b10+
b11+
SDGKISEQSDAK+XL_L
y3+
b5+
b8+
b9+
y10+
